# Blood Pressure in Relation to Concentrations of PCB Congeners and Chlorinated Pesticides

**DOI:** 10.1289/ehp.1002830

**Published:** 2010-11-02

**Authors:** Alexey Goncharov, Marian Pavuk, Herman R. Foushee, David O. Carpenter

**Affiliations:** 1 Department of Environmental Health Sciences, School of Public Health and; 2 Institute for Health and the Environment, University at Albany, Rensselaer, New York, USA; 3 Agency for Toxic Substances and Disease Registry, Atlanta, Georgia, USA; 4 Survey Research Unit, University of Alabama–Birmingham, Birmingham, Alabama, USA

**Keywords:** Ah receptor, Anniston, Alabama, body mass index, hypertension, linear regression

## Abstract

**Background:**

Residents of Anniston, Alabama, live near a Monsanto plant that manufactured polychlorinated biphenyls (PCBs) from 1929 to 1971 and are relatively heavily exposed.

**Objectives:**

The goal of this study was to determine the relationship, if any, between blood pressure and levels of total serum PCBs, several PCB groups with common actions or structure, 35 individual PCB congeners, and nine chlorinated pesticides.

**Methods:**

Linear regression analysis was used to determine the relationships between blood pressure and serum levels of the various contaminants after adjustment for age, body mass index, sex, race, smoking, and exercise in 394 Anniston residents who were not taking antihypertensive medication.

**Results:**

Other than age, total serum PCB concentration was the strongest determinant of blood pressure of the covariates studied. We found the strongest associations for those PCB congeners that had multiple *ortho* chlorines. We found the associations over the full range of blood pressure as well as in those subjects whose blood pressure was in the normal range. The chlorinated pesticides showed no consistent relationship to blood pressure.

**Conclusions:**

In this cross-sectional study, serum concentrations of PCBs, especially those congeners with multiple *ortho* chlorines, were strongly associated with both systolic and diastolic blood pressure.

Blood pressure is regulated by a combination of vessel resistance, neurotransmitter and hormonal control, and other poorly understood factors. Elevated blood pressure is a major risk factor for death from cardiovascular disease and stroke, and incidence increases with age (Rosendorff et al. 2007; Victor 2008). Diet is important, especially reducing intake of salt (Cook et al. 2007) and increasing intake of fruits, vegetables, and nutrients (Appel et al. 2006; Lin et al. 2007). Obesity is believed to be a risk factor for hypertension (Gus et al. 2004; Lin et al. 2009). It has been reported that at least 65 million American adults suffered from hypertension in 1999–2000 (Fields et al. 2004), so understanding of the various factors that alter blood pressure is important.

Some environmental exposures have been found to increase the risk of development of hypertension. There is some modest evidence that smoking increases risk of hypertension (Halperin et al. 2008). Some metals such as arsenic (Chen et al. 2007; Kwok et al. 2007), cadmium (Prozialeck et al. 2008), and lead (Rosin 2009) increase risk of hypertension. Pesticide exposure has been reported to increase risk of hypertensive disorders of pregnancy (Saldana et al. 2009).

There is increasing evidence that chronic exposure to persistent organochlorine contaminants is associated with an elevated prevalence of hypertension (Everett et al. 2008a, 2008b; Ha et al. 2009; Huang et al. 2006). In earlier studies in the Anniston, Alabama, population, we demonstrated a robust relationship between the presence of hypertension (defined as taking antihypertensive medication or having clinical hypertension) and serum polychlorinated biphenyl (PCB) concentrations (Goncharov et al. 2010). The mechanisms responsible for this relationship have not been clearly elucidated. However, endothelial dysfunction has been observed in some studies as a result of exposure to PCBs (Hennig et al. 2002; Toborek et al. 1995).

In this report, we expand on the study of the Anniston population that was the subject of our previous publication on hypertension (Goncharov et al. 2010). We studied whether PCBs alter continuous blood pressure over the full range, including those with “normal” levels. If exposure to these persistent chemicals alters blood pressure even in individuals without disease, the public health implications are even greater. In addition to total PCBs, we examined the association between systolic and diastolic blood pressure and several PCB groups with common actions or structure, individual PCB congeners, and chlorinated pesticides in persons not taking antihypertensive medication, using linear regression analysis. Because we are exploring possible relationships between blood pressure and serum PCB levels, we have excluded those individuals who are taking antihypertensive medication on the assumption that their blood pressure is under control. In this cross-sectional study, we tested the hypotheses that PCB and/or pesticides levels are positively associated with blood pressure and examined whether this is the case with normotensive as well as hypertensive individuals.

## Materials and Methods

The study population consisted of 394 residents of Anniston, Alabama, who lived near the Monsanto plant, who volunteered for study, and who were not taking antihypertensive medication, because such medication will alter blood pressure. This study was reviewed and approved by the institutional review boards of the University at Albany and the University of Alabama–Birmingham, and all study participants provided oral and/or written informed consent before participation in the research study. As previously described (Goncharov et al. 2010), these 394 persons, 18–92 years of age, agreed to be in the study and to provide demographic and medical information including use of medications, smoking, and exercise history. They were measured for height and weight, had fasting blood drawn for serum PCBs, pesticides, and serum lipids, and had three independent measures of blood pressure taken at 2-min intervals after having been seated for 5 min. Because no significant differences were found among the different blood pressure measurements, the average of the three determinations was used for analysis.

Serum PCBs and pesticides (wet weight) were analyzed by the Centers for Disease Control and Prevention’s National Center for Environmental Health laboratory (Atlanta, GA), as previously described (Goncharov et al. 2010). The PCB analysis was of 33 individual congeners and two pairs of coeluting congeners (PCB congeners 28, 44, 49, 52, 66, 74, 87, 99, 101, 105, 110, 118, 128, 138+158, 146, 149, 151, 153, 156, 157, 167, 170, 172, 177, 178, 180, 183, 187, 189, 194, 195, 196 + 203, 199, 206, and 209). The sum of the wet weights of all of these congeners was defined as “total PCBs.” Values below the level of detection (LOD) were taken to be half of the LOD when calculating total PCBs. For the total serum PCB calculation, we included even those congeners that had < 50% of values above the LOD. The relationship between blood pressure and each single congener was determined except for PCB congeners 44, 49, 52, 87, 101, 110, 128, 149, and 151, because these all showed < 50% of measurements above the LOD or had some missing values. The minimum LOD for individual congeners was 2.2 ppt, and the maximum was 63.3 ppt (for PCB-180) for the congeners included in this study. Because of evidence that there is an increased risk of bias when using lipid-adjusted concentrations of PCBs (Schisterman et al. 2005), most analyses were done using wet-weight PCB concentrations while treating serum lipids as a covariate.

Several groups of congeners with either common structural characteristics or common modes of action were evaluated, as described below.

### Estrogen-like congeners (the sum of PCB congeners 44, 49, 66, 74, 99, 110, and 128)

These congeners have estrogenic actions on MCF-7 breast cancer cells (DeCastro et al. 2006).

### Mono-*ortho*-substituted congeners (PCB congeners 28, 66, 74, 105, 118, 156, 157, 167, and 189)

Mono-*ortho* congeners have dioxin-like properties. Six of those PCB congeners (105, 118, 156, 157, 167, and 189) have been assigned a toxic equivalency factor (TEF) of 0.00003 based on a recent evaluation by Van den Berg et al. (2006). We used both the sum of nine mono-*ortho* congener concentrations that we measured and mono-*ortho* PCB toxic equivalents (TEQs; the sum of the six congener concentrations multiplied by their TEFs) in our analyses. We were not able to measure the more potent non-*ortho* (coplanar)-substituted dioxin-like PCB congeners (126, 169, 77, and 81). These generally have concentrations an order of magnitude lower than those of the mono-*ortho* congeners, but their relative contribution to the total dioxin TEQ is higher because their TEFs range from 0.1 to 0.0001.

### Di-, tri-, and tetra-*ortho*-substituted congeners

Di-*ortho*-substituted congeners (the sum of PCB congeners 44, 49, 52, 87, 99, 101, 110, 128, 138+158, 146, 153, 170, 172, 180, and 194) and tri- and tetra-*ortho* congeners (the sum of PCB congeners 149, 151, 177, 178, 183, 187, 195, 196+203, 199, 206, and 209) do not show dioxin-like properties.

We also analyzed nine chlorinated pesticides: hexachlorobenzene, β-hexachlorocyclohexane (β-HCCH), γ-HCCH, oxychlordane, *trans*-nonachlor, *p*,*p*′-dichlorodiphenyldichloroethylene, *o*,*p*′-dichlorodiphenyldichloroethylene, *p*,*p*′-dichlorodiphenyltrichloroethane (*p*,*p*′-DDT), and mirex.

Serum triglycerides and cholesterol were determined in the clinical chemistry laboratory of Jacksonville Medical Center, and total lipid concentrations were calculated using the formula of Bernert et al. (2007):


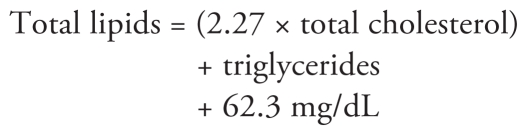


Most analyses were done with total lipids treated as a covariate; but for the sake of comparison with some other published reports, we have included traditional lipid adjustment in one of our tables. In our previous study of the same population, we also analyzed data using cholesterol and triglycerides as separate covariates rather than using the calculated total serum lipids. This resulted in only minor differences in results, and therefore in the present analysis we have used only the more traditional calculated total serum lipid values.

#### Statistical analysis

Initially, we used simple linear regression models, supported by graphical output, to assess crude associations between systolic and diastolic blood pressure and total PCBs without adjustment for any covariate. A similar approach was then used to assess all potential risk factors for elevated blood pressure, including total PCB concentration (in tertiles), body mass index (BMI) in three groups [weight (kilograms)/squared height (meters); categorized as “normal,” < 25 kg/m^2^; “overweight,” 25 to < 30 kg/m^2^; and “obese,” ≥ 30 kg/m^2^], total lipids (in tertiles), sex (male, female), race (Caucasian, African American), smoking status (smoking less than or more than 100 lifetime cigarettes), and exercise (self-report of less than or more than an average of 10 min of daily physical activity). In these models each predictor was adjusted only for age (i.e., without adjustment for the rest of the model covariates), because age was the strongest confounder. Total PCBs and total lipids were used as tertiles to be consistent with our previous analysis where we assessed the same risk factors for hypertension in logistic regression models (Goncharov et al. 2010). Sex, race, smoking, and exercise were included as dichotomous variables. Finally, in multivariate linear regression models we adjusted for BMI and total lipids as continuous variables, and sex (male, female), race (Caucasian, African American), smoking status (smoking less than or more than 100 lifetime cigarettes), and exercise (self-report of less than or more than an average of 10 min of daily physical activity) as dichotomous variables. In addition, we assessed associations between systolic and diastolic blood pressure and total PCBs in those participants with normotensive systolic (mean systolic blood pressure < 140 mmHg) and diastolic (mean diastolic blood pressure < 90 mmHg) blood pressure ranges. Similar adjusted associations were assessed for other toxicants, including 5 PCB groups, 19 individual PCB congeners, and 7 individual pesticides. In these models, all toxicants were analyzed in tertiles. The lowest tertile always served as the reference.

We did not find interactions between total PCBs and any model covariate (age, BMI, total lipids, sex, race, smoking, and exercise) at a *p*-value of < 0.10, which was used in all statistical analyses. After determining the absence of interaction effects, subsequent analysis was performed with one-by-one adjustment for age, BMI, total lipids, sex, race, smoking, and exercise to assess confounding. Those covariates not found to be confounders in these data were still left in the models because they are established risk factors for hypertension.

To assess the presence of multicollinearity among the covariates, we ran the variance inflation factor (VIF) test on all linear regression models. VIF never exceeded the cutoff value 10.

All statistical computations and graphical output were conducted using SAS software (version 9.1.3; SAS Institute Inc., Cary, NC). Each continuous variable was assessed for normality of its distribution both graphically and statistically using the Kolmogorov–Smirnov test. To normalize covariate distributions and their variances, we used logarithmic transformation.

## Results

Table 1 shows results from the population under study, which was 394 residents of Anniston who were not taking antihypertensive medication. For levels of total PCBs, five groups of PCBs with presumed common mechanisms of action, concentrations of individual PCB congeners, and nine chlorinated pesticides measured in this population, see Supplemental Material, Table 1 (doi:10.1289/ehp.1002830).

**Figure 1:**
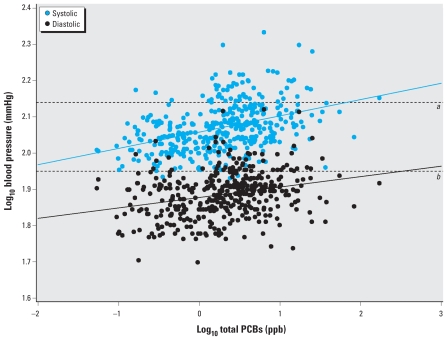
Linear regression of systolic and diastolic blood pressure on total PCB concentration without adjustment for covariates. The dashed lines show cutoff pressures for systolic (*a*) and diastolic (*b*) hypertension. The solid lines show the log of the slope for systolic (β = 0.046) and diastolic (β = 0.029) pressure as a function of the log of the serum PCB concentration.

Figure 1 shows linear regression of the log of systolic and diastolic blood pressure against the log of total PCB concentration for the full population without any adjustment. Of the total population of 394 persons, 341 had systolic blood pressures < 140 mmHg, and 338 had diastolic blood pressures < 90 mmHg. Systolic prehypertension (blood pressure between 120 and 139 mmHg) was present in 116 individuals, diastolic prehypertension (blood pressure between 80 and 89 mmHg) in 112 individuals, and both systolic and diastolic prehypertension in 53 individuals (not included in Table 1). There were 189 persons with normotensive blood pressure. We found a statistically significant relationship between log-transformed blood pressure and log-transformed serum PCB levels for systolic blood pressure (β = 0.046 ± 0.005; *p* < 0.001) and diastolic blood pressure (β = 0.029 ± 0.005; *p* < 0.001). The dashed lines in Figure 1 show the cutoff points for blood pressure levels that define systolic and diastolic hypertension, respectively.

**Figure 2:**
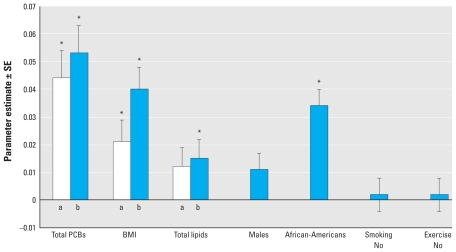
Parameter estimates ± SE of mean diastolic blood pressure in relation to total PCBs and principal risk factors after adjustment for age. For PCBs and total lipids, bars a and b compare the second and third tertile, respectively, with the lowest tertile. For BMI, bars a and b compare overweight and obese categories with the normal-weight category. Sex (here, males) compares males and females; race, African American and Caucasian; smoking, individuals who have not smoked > 100 lifetime cigarettes and those who have; and exercise, individuals who regularly have no more than 10 min of daily exercise and those who do. Although the parameter estimates for males and the second tertile of total lipid concentration are elevated, neither was statistically significant. *p < 0.05.

Figure 2 shows the parameter estimates for mean diastolic blood pressure for each of the major risk factors after adjustment for age and by comparison of second with first and third with first tertiles. Serum PCB levels had the largest parameter estimate of all covariates, and we found little difference between the second and third tertiles, suggesting that this is a low-dose effect. BMI was important, and the parameter estimate increased with increasing BMI. However, the obese category for the BMI parameter estimate was not greater than the second tertile of PCB concentration. Total lipids and race were also significant, but sex, smoking, and exercise were not.

Table 2 shows linear regression of total serum PCBs in relation to systolic and diastolic blood pressure for the full population (*n* = 394) and for those whose blood pressure was within the “normotensive” range (systolic < 140 mmHg, *n* = 341; diastolic < 90 mmHg, *n* = 338) after adjustment for all covariates. Both the second and third tertiles were significant for the full range of systolic and diastolic blood pressures whether or not PCB concentrations were lipid adjusted. For those whose blood pressure was in the normotensive range, we observed a significant relationship for third versus first tertiles for both systolic and diastolic pressure and a significant relation for diastolic but not systolic pressure for second versus first tertile. However, the low-dose effect was more obvious when considering the full range of blood pressures than for those who were normotensive. We found little difference when lipids were treated as a covariate or lipid-adjusted PCB concentrations were used.

Table 3 presents multiple regression analysis of log-transformed mean systolic and diastolic blood pressure in relation to total PCB concentration and five congener groups after adjustment for all other covariates. For detailed information on the concentrations of individual congeners and congener groups, see Supplemental Material, Tables 1 and 2 (doi:10.1289/ehp.1002830). Both systolic and diastolic blood pressures were significantly correlated with total serum PCBs, di-*ortho* congeners, and tri- plus tetra-*ortho* congeners when both the second and third tertiles were compared with the first tertile. Estrogen-like and mono-*ortho* congeners (sum of concentrations, not TEQs) were not significantly correlated with either systolic or diastolic blood pressure. Mono-*ortho* PCB TEQs were not significantly related to blood pressure, although they were close to being significant for diastolic pressure. However, because we did not measure the more potent dioxin-like PCBs (non-*ortho*-substituted PCB congeners 77, 81, 126, and 169), the significance of this result is uncertain.

Table 4 shows the linear regression analysis for those individual PCB congeners that showed significant relationships with blood pressure in the full population. The mono-*ortho* congeners 156, 157, and 189 showed a significant relationship; all these congeners have an assigned TEF. We observed the strongest association with PCB congeners 170, 172, 180 (all di-*ortho*), 178, 183, 187, 196+203, and 199 (all tri- or tetra-*ortho*). For all of these congeners, we found a significant relationship between concentrations and both systolic and diastolic blood pressure in both the second and third tertiles of PCBs. Supplemental Material, Table 2 (doi:10.1289/ehp.1002830) provides the same information on those congeners that did not show statistically significant associations.

Table 5 shows multiple linear regression analysis of mean systolic and diastolic blood pressure by tertiles of levels of pesticides. We observed no consistent significant association for chlorinated pesticides.

## Discussion

Our results show that serum PCB concentration is more strongly associated with elevation of blood pressure than with any other factor except age, and that this relationship applies both to those individuals who are not taking antihypertensive medication and to the subset of persons whose blood pressure is within the normotensive range. We have previously reported that serum PCB levels are significant risk factors for hypertension using logistic regression analysis (Goncharov et al. 2010), and the present results show that the association is found over any range of blood pressure. The association appears to be specific to PCBs, because we found little or no relationship between blood pressure and levels of organochlorine pesticides.

The relationship with blood pressure was strongest with *ortho*-substituted PCB congeners with two or more chlorines. Although we found no significant relationship for the sum of all mono-*ortho* congeners, we found weak but significant associations for three mono-*ortho* congeners, all of which have TEF values. Unfortunately, we did not have measurements of the most potent non-*ortho* dioxin-like PCB congeners. We found no evidence for a relationship between estrogenic congeners as identified by DeCastro et al. (2006) and blood pressure.

Anniston residents have relatively high levels of serum PCBs. It is difficult to compare levels of total PCBs across different studies because investigators analyze for different congeners, and some report wet-weight and others lipid-adjusted values. Needham et al. (2005) and the 2003–2004 National Health and Nutrition Examination Survey (NHANES) report (Centers for Disease Control and Prevention 2009) provide levels of some single congeners, and, of the congeners common to our study, levels of PCB congeners 99, 118, 153, 156, 170, 180, and 183 in our subjects are greater than those of the 90th percentile of levels in the general U.S. population. Only the level of PCB-74 was lower in this sample of Anniston residents than that reported for the 90th percentile of the general U.S. population. This is an interesting congener that has often been found to reflect fish consumption (Fitzgerald et al. 2004).

There are limitations to our study. By its cross-sectional nature, this study cannot prove causality no matter how strong the associations. There also are some limitations in information that should be considered, such as diet and better measures of levels of exercise and smoking.

There has been some evidence for an association of elevated serum PCBs with blood pressure since the study by Kreiss et al. (1981) of residents of Triana, Alabama, who were exposed by consuming fish downstream from an industrial facility. In a study of PCB exposure from living in the vicinity of waste sites, Stehr-Green et al. (1986) found a dose-dependent but not statistically significant relationship between serum PCB levels and high blood pressure. Huang et al. (2006) found elevated rates of hospitalization for hypertension among residents of New York who lived near hazardous waste sites containing persistent organic pollutants, primarily PCBs.

Two recent investigations have used the 1999–2002 NHANES to determine the relationship between exposure to various persistent organic pollutants and diagnosed hypertension. Ha et al. (2009) reported that the sum of three dioxins and three furans showed significant elevations in rates of hypertension in women, whereas serum PCBs tended toward positive associations in men. Everett et al. (2008a) investigated associations between hypertension and individual PCB congeners and found the strongest associations with dioxin-like PCBs (PCBs 126 and 118) but also found significant relationships with PCB congeners 74, 99, 138+158, 170, and 187. Everett et al. (2008b) expanded their study to include NHANES data through 2004 and found significant associations with PCB congeners 74, 118, and 126. In the expanded data set of Everett et al. (2008b), the associations for PCB congeners 99, 138+158, 153, 156, 169, 170 180, and 187 were elevated but not statistically significant. In a study focused on the metabolic syndrome in the Japanese population, Uemura et al. (2009) reported significant associations between hypertension and PCB congeners 105, 114, 118, 123, 126, and 167 but not with PCB congeners 156, 157, 169, or 189. They also found significant associations with levels of four dioxins and two furans.

Our results are in general agreement with those of these previous studies, even though in this study we are investigating blood pressure rather than hypertension and not all studies measured the same PCB congeners. Despite not monitoring the most potent dioxin-like congeners, we did find significant associations with levels of PCBs 156, 157, and 189, all of which have some dioxin-like activity. Although we found significant associations between blood pressure and some 20 congeners, we observed a clear pattern of stronger associations with those congeners with multiple *ortho* chlorines. Of these, PCB congeners 170, 172, 178, 180, 183, 187, 196+203, and 199 showed statistically significant associations in both the second and third tertile with both systolic and diastolic blood pressure. Our results differ somewhat from the conclusions above in that we found a dominance of associations with multi-*ortho*-substituted congeners, not dioxin-like mono-*ortho* congeners. It is also striking that the association between serum PCB levels and blood pressure appears to be a low-dose effect, in that we found little difference between the parameter estimates when comparing the first and second tertiles versus comparing the first and third tertiles in all those not on antihypertensive medication (Figure 2, Table 2). This is less apparent in normotensive individuals. If these associations are indeed present at relatively common serum PCB concentrations, the public health significance of the relationships may be considerable. Clearly, it is very important that these possibilities be tested systematically in a prospective investigation. There also is a need for investigations that can identify the mechanisms that might underlie these associations.

We found little if any association between blood pressure and levels of chlorinated pesticides. Siddiqui et al. (2002) reported an association between maternal blood pressure and levels of γ-HCCH and *p*,*p*′-DDT in breast milk. However, overall our results do not support a major role for chlorinated pesticides in regulation of blood pressure.

The finding that the association between serum PCB levels and blood pressure is present in individuals with normal blood pressure is important and raises the important question of what is “normal.” This observation implies that even background levels of PCBs are altering physiological processes and suggests that there is no threshold for action. Our previous studies in a Native American population showed relationships between serum PCB levels and “normal” levels of thyroid hormone (Schell et al. 2008) and testosterone (Goncharov et al. 2009), and also performance on cognitive tests in a “normal” range in adolescents (Newman et al. 2009) and adults (Haase et al. 2009). For these physiological effects, as with blood pressure, a number of external factors can influence levels or performance, but the observation that serum PCB levels can influence them indicates that PCB levels may cause physiological changes and contribute to the development of diseases even when they are not the sole cause.

## Conclusion

Hypertension, like diabetes and cardiovascular disease, is often considered a “lifestyle” disease that is the result of stress, diet, and lack of exercise. Inadequate attention has been paid to the role of exposure to environmental chemicals. Our results suggest that, in addition to some metals, PCBs and possibly other persistent organic pollutants (but not chlorinated pesticides) may be important factors both in the development of hypertension and in setting the “normal” blood pressure.

## Figures and Tables

**Table 1:** Characteristics of the study participants (*n* = 394).^a^

Covariate	Median	Mean ± SD	Range	Proportion
Mean systolic blood pressure (mmHg)^b^	117.0	119.1 ± 19.04	85.3–215.3	—
Mean diastolic blood pressure (mmHg)^b^	78.3	77.7 ± 11.71	50.0–132.0	—
Total PCBs (ng/g)	2.12	4.72 ± 11.05	0.09–170.42	—
Age (years)	47.0	47.6 ± 15.52	18.0–92.0	—
BMI (kg/m^2^)	28.0	29.7 ± 7.24	18.0–65.0	—
Total lipids^c^ (mg/L)	605.0	629.1 ± 154.36	335.8–1436.2	—
Sex (*n* = 126 males, 268 females)^d^	—	—	—	1:2.12
Race (*n* = 171 blacks, 223 whites)^e^	—	—	—	1:1.30
Smoking (*n* = 173 no, 220 yes)^f^	—	—	—	1:1.27
Exercise (*n* = 171 no, 221 yes)^g^	—	—	—	1:1.29

a Sample size may be less than shown for some characteristics because of missing data.

b Mean values were calculated from three independent blood pressure measurements.

c Estimated total lipids based on direct measurement of serum total cholesterol and triglycerides.

d 0 = males, 1 = females.

e 0 = whites, 1 = blacks.

f 0 = nonsmokers, 1 = smoked > 100 cigarettes during lifetime.

g 0 = no exercise, 1 = daily moderate physical exercise of at least 10 min duration.

**Table 2:** Linear regression of log-transformed blood pressure in relation to total wet-weight and lipid-adjusted PCB concentration.

	Second versus first tertile		Third versus first tertile	
Group	β ± SE	*p*-Value	β ± SE	*p*-Value
Full (wet weight)				

Systolic	0.023 ± 0.09	0.0088	0.028 ± 0.10	0.0092
Diastolic	0.034 ± 0.09	0.0002	0.035 ± 0.10	0.0011

Full (lipid adjusted)				

Systolic	0.030 ± 0.008	0.01	0.032 ± 0.01	0.006
Diastolic	0.033 ± 0.008	0.0002	0.037 ± 0.10	0.0005

Normotensive (wet weight)				

Systolic	0.007 ± 0.006	0.57	0.020 ± 0.007	0.035
Diastolic	0.013 ± 0.007	0.06	0.020 ± 0.008	0.04

Normotensive (lipid adjusted)				

Systolic	0.011 ± 0.006	0.075	0.023 ± 0.007	0.002
Diastolic	0.018 ± 0.007	0.012	0.026 ± 0.008	0.003

Data are adjusted for all other model covariates for the subject population (full; *n* = 394) and for those with normotensive systolic (*n* = 341) and diastolic (*n* = 338) blood pressures (normotensive). β-Coefficients (adjusted for age, BMI, total lipids, sex, race, smoking, and physical activity) are presented by considering serum PCB concentration by tertiles. Total wet-weight PCB concentration in tertiles: first (referent), 0.092–1.23 ppb; second, 1.24–3.65 ppb; third, 3.66–170.42 ppb. Total lipid-adjusted PCBs concentration in tertiles: first (referent), 14.36–201.6 ng/g lipids; second, 201.7–557.0 ng/g lipids; third, 557.1–26649.0 ng/g lipids.

**Table 3:** Multiple linear regression analysis of logarithmically transformed mean systolic and diastolic blood pressure (*n* = 394) by PCB concentration.

	Second versus first tertile		Third versus first tertile	
Toxicant	β ± SE	*p*-Value	β ± SE	*p*-Value
Systolic blood pressure				

Total PCBs	0.024 ± 0.009	0.008	0.031 ± 0.01	0.009
Estrogen-like	0.003 ± 0.008	0.53	0.015 ± 0.009	0.11
Mono-*ortho*	0.011 ± 0.009	0.16	0.013 ± 0.01	0.27

PCB TEQs				

Mono-*ortho*	0.00058 ± 0.009	0.91	0.002 ± 0.010	0.88
Di-*ortho*	0.019 ± 0.009	0.028	0.027 ± 0.011	0.019
Tri- and tetra-*ortho*	0.024 ± 0.009	0.0057	0.043 ± 0.01	0.0003

Diastolic blood pressure				

Total PCBs	0.032 ± 0.009	0.0002	0.033 ± 0.01	0.0011
Estrogen-like	0.005 ± 0.008	0.27	0.011 ± 0.009	0.11
Mono-*ortho*	0.016 ± 0.009	0.06	0.018 ± 0.01	0.07

PCB TEQs				

Mono-*ortho*	0.004 ± 0.009	0.30	0.00045 ± 0.010	0.63
Di-*ortho*	0.024 ± 0.009	0.0025	0.028 ± 0.009	0.0044
Tri- and tetra-*ortho*	0.026 ± 0.009	0.0011	0.043 ± 0.010	0.0014

Data are for effects of total PCBs and PCB TEQs, in tertiles, after adjustment for age, BMI, total lipids, sex, race, smoking status, and physical activity.

**Table 4:** Multiple linear regression analysis of log-transformed mean systolic and mean diastolic blood pressure for the full population (*n* = 394) on tertiles of individual PCB congeners.

	Second versus first tertile		Third versus first tertile	
Toxicant	β ± SE	*p*-Value	β ± SE	*p*-Value
Mono-*ortho* PCB congeners				

PCB-156				

Systolic	0.018 ± 0.009	0.04	0.022 ± 0.011	0.04
Diastolic	0.028 ± 0.009	< 0.01	0.030 ± 0.011	< 0.01

PCB-157				

Systolic	0.015 ± 0.008	0.07	0.020 ± 0.010	0.04
Diastolic	0.022 ± 0.009	0.01	0.027 ± 0.010	0.01

PCB-189				

Systolic	0.009 ± 0.008	0.27	0.029 ± 0.010	< 0.01
Diastolic	0.014 ± 0.009	0.05	0.030 ± 0.010	< 0.01

Di-*ortho* PCB congeners				

PCB-138+158				

Systolic	0.014 ± 0.008	0.09	0.017 ± 0.010	0.04
Diastolic	0.021 ± 0.008	0.01	0.023 ± 0.010	0.02

PCB-146				

Systolic	0.013 ± 0.008	0.01	0.020 ± 0.010	0.04
Diastolic	0.018 ± 0.008	0.04	0.024 ± 0.010	0.01

PCB-153				

Systolic	0.015 ± 0.008	0.07	0.021 ± 0.010	0.03
Diastolic	0.026 ± 0.008	< 0.01	0.033 ± 0.010	< 0.01

PCB-170				

Systolic	0.023 ± 0.009	< 0.01	0.031 ± 0.011	< 0.01
Diastolic	0.031 ± 0.009	< 0.01	0.037 ± 0.011	< 0.01

PCB-172				

Systolic	0.020 ± 0.009	0.03	0.031 ± 0.011	< 0.01
Diastolic	0.026 ± 0.009	< 0.01	0.033 ± 0.011	< 0.01

PCB-180				

Systolic	0.025 ± 0.009	< 0.01	0.030 ± 0.011	< 0.01
Diastolic	0.030 ± 0.009	< 0.01	0.036 ± 0.011	< 0.01

PCB-194				

Systolic	0.016 ± 0.009	0.06	0.031 ± 0.011	< 0.01
Diastolic	0.025 ± 0.009	< 0.01	0.033 ± 0.011	< 0.01

Tri-and tetra-*ortho* PCB congeners				

PCB-177				

Systolic	0.012 ± 0.008	0.13	0.019 ± 0.009	0.04
Diastolic	0.020 ± 0.008	0.02	0.025 ± 0.009	0.01

PCB-178				

Systolic	0.021 ± 0.009	0.02	0.027 ± 0.010	0.01
Diastolic	0.032 ± 0.009	< 0.01	0.037 ± 0.010	< 0.01

PCB-183				

Systolic	0.022 ± 0.008	< 0.01	0.024 ± 0.009	0.01
Diastolic	0.025 ± 0.008	< 0.01	0.030 ± 0.010	< 0.01

PCB-187				

Systolic	0.027 ± 0.008	< 0.01	0.031 ± 0.010	< 0.01
Diastolic	0.032 ± 0.008	< 0.01	0.040 ± 0.010	< 0.01

PCB-195				

Systolic	0.010 ± 0.008	0.21	0.024 ± 0.010	0.01
Diastolic	0.020 ± 0.008	0.02	0.030 ± 0.010	< 0.01

PCB-196+203				

Systolic	0.026 ± 0.009	< 0.01	0.042 ± 0.011	< 0.01
Diastolic	0.035 ± 0.009	< 0.01	0.043 ± 0.011	< 0.01

PCB-199				

Systolic	0.023 ± 0.009	0.01	0.034 ± 0.011	< 0.01
Diastolic	0.032 ± 0.009	< 0.01	0.037 ± 0.011	< 0.01

PCB-206				

Systolic	0.015 ± 0.009	0.09	0.028 ± 0.011	0.01
Diastolic	0.026 ± 0.009	< 0.01	0.027 ± 0.011	0.02

PCB-209				

Systolic	0.016 ± 0.009	0.07	0.030 ± 0.010	0.01
Diastolic	0.028 ± 0.009	< 0.01	0.038 ± 0.012	< 0.01

Data are adjusted for age, BMI, total lipids, sex, race, smoking, and physical activity. Only statistically significant congeners have been included.

**Table 5:** Multiple linear regression analysis of mean systolic and mean diastolic blood pressure (*n* = 394) on tertiles of pesticides after adjustment for age, BMI, total lipids, sex, race, smoking, and physical activity.

	Second versus first tertile		Third versus first tertile	
Toxicant	β ± SE	*p*-Value	β ± SE	*p*-Value
HCB				

Systolic	−3.4 × 10^−3^ ± 0.007	0.64	−1.7 × 10^−4^ ± 0.009	0.98
Diastolic	3.7 × 10^−3^ ± 0.007	0.62	9.9 × 10^−3^ ± 0.009	0.91

β-HCCH				

Systolic	2.4 × 10^−3^ ± 0.010	0.30	8.4 × 10^−3^ ± 0.008	0.82
Diastolic	3.5 × 10^−3^ ± 0.010	0.74	0.016 ± 0.008*	0.018

γ-HCCH				

Systolic	2.7 × 10^−3^ ± 0.007	0.70	9.0 × 10^−3^ ± 0.007	0.22
Diastolic	−4.3 × 10^−3^ ± 0.007	0.54	4.4 × 10^−3^ ± 0.007	0.56

Oxychlordane				

Systolic	2.3 × 10^−3^ ± 0.007	0.77	3.5 × 10^−3^ ± 0.010	0.72
Diastolic	9.1 × 10^−3^ ± 0.008	0.26	−1.1 × 10^−3^ ± 0.010	0.88

*trans*-Nonachlor				

Systolic	8.1 × 10^−3^ ± 0.007	0.27	9.1 × 10^−3^ ± 0.009	0.36
Diastolic	0.014 ± 0.007	0.07	7.5 × 10^−3^ ± 0.009	0.43

*p*,*p*′-DDE				

Systolic	−5.3 × 10^−3^ ± 0.008	0.45	7.4 × 10^−3^ ± 0.009	0.46
Diastolic	5.5 × 10^−3^ ± 0.008	0.53	3.4 × 10^−3^ ± 0.009	0.73

*o*,*p*′-DDT				

Systolic	1.1 × 10^−3^ ± 0.007	0.81	1.5 × 10^−3^ ± 0.008	0.13
Diastolic	2.2 × 10^−3^ ± 0.007	0.70	3.1 × 10^−3^ ± 0.007	0.66

*p*,*p*′-DDT				

Systolic	8.7 × 10^−3^ ± 0.007	0.90	1.3 × 10^−2^ ± 0.008	0.87
Diastolic	6.8 × 10^−2^ ± 0.007	0.35	−1.7 × 10^−2^ ± 0.008	0.82

Mirex				

Systolic	7.5 × 10^−3^ ± 0.008	0.35	8.1 × 10^−3^ ± 0.010	0.45
Diastolic	2.0 × 10^−2^ ± 0.008	0.06	1.8 × 10^−2^ ± 0.010	0.07

Abbreviations: DDE, dichlorodiphenyldichloroethylene; HCB, hexachlorobenzene.

* *p* < 0.05.
